# Supplementary material for: Can participatory budgeting mitigate government debt risk？—An empirical analysis using cross-national panel data
Source: PLoS One. 2025 Dec 8;20(12):e0324411. doi: 10.1371/journal.pone.0324411 (PMC12685174; doi:10.1371/journal.pone.0324411)
Supplement: Attachment — Submitted filename: Response_to_Reviewers_auresp_2.docx [file pone.0324411.s004.docx]

**Response to Reviewers**

**1.The authors could clarify the aim by pointing out to the realistic scope of PB’s influence — distinguishing between local participatory processes and national fiscal management systems.**

**Our response:**

We extend our sincere gratitude to the reviewer for their insightful comments, which rightly point to an issue that we have been continuously attentive to in our research but had not yet fully articulated in this paper. As the reviewer rightly notes, the existing literature on participatory budgeting has predominantly focused on practices at the local level, with relatively little attention paid to its application at the national level.

Specifically, participatory budgeting at the local government level—typically carried out in municipalities, counties, and other sub-national administrative tiers—centers on budget allocation decisions within specific jurisdictions, such as community projects or infrastructure development. It is characterized by a relatively narrow scope of participation and tends to address localized, concrete budgetary matters.

In contrast, this study examines participatory budgeting at the national level, primarily drawing on the International Budget Partnership's Open Budget Survey. Our focus is on budget transparency and public participation mechanisms at the national level, assessing the extent of budget disclosure by the central government, the channels for public engagement, and the development of accountability systems. This approach offers a broader research perspective, encompassing central budget policies, fiscal reporting systems, and the feedback mechanisms of national audit results, among other dimensions.

While participatory budgeting at the local and national levels differs in terms of the scope and process of participation, they share fundamental common ground in their core objectives and can form complementary linkages. These shared goals are mainly reflected in three aspects: first, both aim to promote budget democratization, enhance public voice in fiscal decision-making, and thereby achieve more efficient and equitable allocation of public resources; second, both emphasize "collaborative governance," striving to improve fiscal transparency, deepen public participation, and strengthen government accountability.

At the practical level, the two form a mutually reinforcing virtuous cycle: local practices provide valuable experiential foundations for institutionalized participation at the national level, while IBP’s national-level assessments help create a favorable macro-environment for deeper local engagement by promoting legal framework reforms and data disclosure. Specifically, countries with high scores in the IBP survey tend to be more supportive of the expansion of local participatory budgeting; conversely, successful local cases can also enhance a country’s performance in IBP evaluations. At the same time, the gaps in national-level participation revealed by the IBP survey can motivate local governments to introduce or improve relevant practices.

Accordingly, this study adopts the score for "public participation in the budget process" from the International Budget Partnership's Open Budget Survey (OBS) as a proxy variable to measure a country's level of participatory budgeting (PB) practice, with the aim of empirically examining whether and how citizen engagement in public budgeting affects government debt risk. In terms of theoretical construction, the study does not strictly distinguish between local and national levels of citizen participation but conceptualizes it as a macro-level, holistic institutional factor, seeking to analyze the relationship between citizen budget participation and fiscal risk from a broader perspective.

The above modifications have been highlighted in the manuscript (lines 79–101) for clarity.

**2.The introduction section could engage with more literature, e.g. Džinić, J., et al. 2016. Participatory budgeting: a comparative study of Croatia, Poland and Slovakia. NISPAcee Journal of Public Administration and Public Policy, vol. IX, no. 1, p. 31 - 56.; Murray Svidroňová, M., Benzoni Baláž, M., Klimovský, D. and Kaščáková, A. (2024), "Determinants of sustainability of participatory budgeting: Slovak perspective", Journal of Public Budgeting, Accounting & Financial Management, Vol. 36 No. 1, pp. 60-80.; Murray Svidroňová, M., Nikolov, M., Garvanlieva Andonova, V., & Kaščáková, A. (2023). COVID-19 and participatory budgeting in North Macedonia and Slovakia. Public Sector Economics, 47(3), 387-406.**

**Our response:**

We sincerely thank the reviewer for their insightful comments. We fully agree with the reviewer’s observation that the introduction would benefit from additional references to better frame the research problem. We are pleased to have incorporated two of the three literature recommendations provided by the reviewer. In addition, to further strengthen the research context, we have supplemented the text with three additional relevant references. In total, this round of revisions has added five new references to the introduction. The specific changes can be found in lines 49–53, 63–65, and 68–74 of the manuscript, and the newly included references are listed as items [2], [9], and [11–13] in the reference list.

**3.Also, clarify the conceptual boundaries of “participatory budgeting” at national level.**

**Our response:**

We thank the reviewer for their valuable comments. We agree with the reviewer’s observation that there are indeed differences in the specific scope and processes of participation between traditional forms of participatory budgeting and the model examined in this paper. Nevertheless, the two approaches converge in their fundamental principles and ultimate objectives—namely, both are committed to promoting the rational allocation of budgetary resources and enhancing fiscal transparency.

Taking the 2023 survey as an example, it included a total of 18 questions related to public participation in the budgeting process, numbered from 125 to 142, which specifically cover:

（1）Does the executive use participation mechanisms through which the public can provide input during the formulation of the annual budget (prior to the budget being tabled in parliament)?

（2）With regard to the mechanism identified in question 125, does the executive take concrete steps to include vulnerable and under-represented parts of the population in the formulation of the annual budget?

（3）"During the budget formulation stage, which of the following key topics does the executive’s engagement with citizens cover?（including：1. Macroeconomic issues； 2. Revenue forecasts, policies, and administration； 3. Social spending policies； 4. Deficit and debt levels； 5. Public investment projects； 6. Public services）

（4）Does the executive use participation mechanisms through which the public can provide input in monitoring the implementation of the annual budget?

（5）With regard to the mechanism identified in question 128, does the executive take concrete steps to receive input from vulnerable and underrepresented parts of the population on the implementation of the annual budget?

（6）"During the implementation of the annual budget, which of the following topics does the executive’s engagement with citizens cover?

（7）"When the executive engages with the public, does it provide comprehensive prior information on the process of the engagement, so that the public can participate in an informed manner?

（8）With regard to the mechanism identified in question 125, does the executive provide the public with feedback on how citizens’ inputs have been used in the formulation of the annual budget?

（9）With regard to the mechanism identified in question 128, does the executive provide the public with information on how citizens’ inputs have been used to assist in monitoring the implementation of the annual budget?

（10）Are participation mechanisms incorporated into the timetable for formulating the Executive’s Budget Proposal?

（11）Do one or more line ministries use participation mechanisms through which the public can provide input during the formulation or implementation of the annual budget?

（12）Does the legislature or the relevant legislative committee(s) hold public hearings and/or use other participation mechanisms through which the public can provide input during its public deliberations on the formulation of the annual budget (pre-budget and/or approval stages)?

（13）"During the legislative deliberations on the annual budget (pre-budget or approval stages), which of the following key topics does the legislature’s (or relevant legislative budget committee) engagement with citizens cover?

（14）Does the legislature provide feedback to the public on how citizens’ inputs have been used during legislative deliberations on the annual budget?

（15）Does the legislature hold public hearings and/or use other participation mechanisms through which the public can provide input during its public deliberations on the Audit Report?

（16）Does the Supreme Audit Institution (SAI) maintain formal mechanisms through which the public can suggest issues/topics to include in the SAI’s audit program (for example, by bringing ideas on agencies, programs, or projects that could be audited)?

（17）Does the Supreme Audit Institution (SAI) provide the public with feedback on how citizens’ inputs have been used to determine its audit program?

（18）Does the Supreme Audit Institution (SAI) maintain formal mechanisms through which the public can contribute to audit investigations (as respondents, witnesses, etc.)?”

As evidenced by the survey questions above, the International Budget Partnership's Open Budget Survey demonstrates substantive alignment with traditional participatory budgeting in its operational approach to assessing opportunities for public participation in the budget process. To address the reviewer's comment and enhance conceptual precision, we have provided a theoretical clarification of "national-level participatory budgeting" in the revised manuscript. The specific modifications can be found in lines 78-87 of the text.

**4.The econometric specifications are standard and competently applied, though results should be interpreted as correlational with causal tendencies, not definitive causal proof.**

**Our response:**

We gratefully acknowledge the reviewer for raising this important point. We agree that the estimates from the fixed-effects model reflect the average trends within the sample. Accordingly, we have revised the relevant wording in the manuscript to present our findings more precisely. The specific changes can be found in lines 27–28 of the abstract and lines 583–586 of the conclusion.

**5.For future research consider multilevel modelling if future data allow (subnational PB vs. national debt outcomes).**

**Our response:**

We appreciate the reviewer's astute methodological recommendation. The suggestion to employ multilevel modelling is well-noted. Indeed, this approach would be highly appropriate for capturing the hierarchical structure of the data (e.g., subnational entities nested within countries over time), allowing for a more nuanced analysis of how subnational public borrowing (Level 1) influences national debt outcomes (Level 2), while accounting for unobserved heterogeneity at both levels. While our current dataset does not possess the necessary breadth or depth of subnational data to robustly implement this method, we fully acknowledge its potential. We have explicitly highlighted the consideration of multilevel modelling as a key recommendation for future studies in our revised discussion section.

In response to the reviewer's suggestion, we have added a third future research (3) to the "Limitations and Scope for Future Research" section. The specific changes can be found in lines 630-635 of the revised manuscript.
